# Is Elite Sport (Really) Bad for You? Can We Answer the Question?

**DOI:** 10.3389/fpsyg.2017.00324

**Published:** 2017-03-03

**Authors:** Florence Lebrun, Dave Collins

**Affiliations:** Institute of Coaching and Performance, School of Sport and Wellbeing, University of Central LancashirePreston, UK

**Keywords:** clinical diagnosis, (dys)functional, elite performers, genesis, mental health

## Abstract

Elite athletes are not immune to mental health issues. Yet, quality research on mental health in elites has so far been limited. Thus, while research on mental health emphasises the prevalence and nature of disorders in the general population, its extent in elite performers remains unclear. Indeed, the prevalence of mental conditions cannot be accurately calculated in elite athletes due to a lack of diagnostic criteria and screening tools specifically adapted to this unique population. Researchers and practitioners are, therefore, confronted with biases reflecting the use of clinical norms and instruments initially developed for the general population. Furthermore, without considering the athlete persona as well as the sport culture in which elites play, there is a risk of under- or over-estimating the prevalence of mental health issues in high-performance environments. Due to the unique characteristics surrounding an elite athlete’s life, we therefore suggest a change of perspective: moving from the usual normal-versus-pathological to a functional-versus-dysfunctional approach. Implications for future research and practice are discussed, most notably examining practitioners’ expertise in diagnosing and treating elite performers.

## Introduction

Concerns about mental health have been growing for years (World Health Organization [WHO], 2016) and reached the sports world where research on elite performers has recently experienced a sudden development. Contrary to previous common assumption, elite athletes also suffer from mental disorders, such as depression, anxiety, eating disorders, to name a few (Markser, 2011; Hughes and Leavey, 2012; Gouttebarge et al., 2015a,b). A frequent question, however, is whether elite athletes—often perceived by the public as *super*-humans—are more at risk of mental health issues than the general population? Research evidence on this topic is, so far, inconclusive and debatable. On one hand, studies have suggested that the prevalence of mental health issues in sport is comparable to the general population (Markser, 2011; Schaal et al., 2011; Gulliver et al., 2015; Rice et al., 2016). On the other, research has argued that elite athletes might be more at-risk to develop certain mental health issues (Roberts et al., 2016) such as eating disorders (Byrne and McLean, 2002; Sudi et al., 2004; Bär and Markser, 2013; Thompson and Sherman, 2014), depression (Hughes and Leavey, 2012), and common mental disorders (i.e., distress, anxiety, depression, substance abuse; Gouttebarge et al., 2015a,b). There is, therefore, a rationale for further investigation into the mental health of *elite* level athletes.

Unfortunately, however, there is an overall lack of quality research investigating mental health in elite sport (Rice et al., 2016; Sebbens et al., 2016). This dearth results, at least in part, from a lack of consensus regarding the diagnosis of mental disorders in elites as well as from a lack of psychometric measures specifically adapted to this peculiar population. In the absence of such a specific focus (Reardon and Factor, 2010; Markser, 2011; Bär and Markser, 2013; Rice et al., 2016), the prevalence and nature of mental health issues faced cannot yet be precisely estimated (Doherty et al., 2016; Roberts et al., 2016). Therefore, the assumption that the prevalence of mental health disorders is comparable in elite athletes than in the general population (Markser, 2011; Schaal et al., 2011; Gulliver et al., 2015) seems hasty.

Compared to Mr. and Ms. Everybody, elite athletes have to face additional sport-related stressors and physical demands throughout their entire sporting career (Hughes and Leavey, 2012; MacNamara and Collins, 2015). Therefore, since the general population may never have to deal with the types of pressure encountered in high performance environments, can the comparison between elite athletes and normal people be considered as fair? Given the differences in both nature and challenge, are we guilty of comparing apples and oranges? Also, whilst conditions such as common mental disorders (Gouttebarge et al., 2015a,b), anxiety disorders (Markser, 2011), depression (Hughes and Leavey, 2012; Armstrong et al., 2015; Doherty et al., 2016) and eating disorders (Byrne and McLean, 2002; Sudi et al., 2004; Bär and Markser, 2013; Thompson and Sherman, 2014) have been intensively studied, others have been overlooked in the context of high performance (e.g., psychotic disorders, bipolar disorders, phobia, ADHD, behavioural disorders; Reardon and Factor, 2010; Markser, 2011; Bär and Markser, 2013; Rice et al., 2016). Consequently, conclusions on the prevalence of mental health disorders in elite athletes in comparison with the general population (Markser, 2011; Schaal et al., 2011; Gulliver et al., 2015) may be biased and, and as previously mentioned, somewhat premature.

Reflecting these concerns, this paper aims to address three particular issues relating to mental health in elite sport that seem to have been so far overlooked: (i) differences in the genesis of conditions; (ii) the challenges of applying general population science to non-ordinary people such as elite athletes, and; (iii) the functionality or dysfunctionality of behaviours depending on the context in which they occur. Ensuing from those concerns, we discuss the implications for researchers and mental health professionals working in high-performance environments.

## Person Versus Athlete: The Genesis of Mental Health Issues

It seems sensible to discriminate between issues based on their cause. In short, are the issues occurring because the individual is an athlete or for some other, perhaps biological reason that would have led to problems irrespective of their career choice (Martindale et al., 2014)? Injuries, competitive pressure, psychosocial stressors, performance issues, and getting older have all been shown to represent a risk for elite athletes to develop a mental disorder such as depression (Reardon and Factor, 2010; Hughes and Leavey, 2012; Rice et al., 2016). Periods of transition, especially retirement from sport, appear to be another at-risk period for psychological distress in elite athletes (Reardon and Factor, 2010; Hughes and Leavey, 2012). Nevertheless, Reardon and Factor (2010) have rightly pointed out that “athletes’ depression might have nothing to do with their athletic pursuits or the athletic pursuits could be their way of coping with depression, or it even could be caused by athletic participation. These possibilities have not been studied *per se*” (p. 963). In short, both the genesis and underlying reasons for maintenance need to be considered when examining an elite athlete issue.

Adding to this complexity, the reasons behind the development of a mental disorder can be different from one person to another and therefore, from one athlete to another (Klinkowski et al., 2008). Factors such as the sport type and related demands (e.g., potential link between depression of clinical severity and frequent concussions; cf. Guskiewicz et al., 2007), skill level, age, gender, wage, media coverage, etc., need to be considered when comparing the prevalence of mental issues from one sport to another (Schaal et al., 2011; Arnold and Fletcher, 2012; Rice et al., 2016). Likewise, the performer’s personal and familial medical history as well as the possibility of a chemical or hormonal imbalance in the brain (e.g., depression) should be investigated before jumping to any conclusion letting us believe that elite sport participation is causing the development of a mental disorder. Before making a diagnosis, the individual, the athlete persona, and the context (i.e., familial, sport, and social context) need to be carefully examined (cf. the Biopsychosocial approach – Collins et al., 2012). In short, following Reardon and Factor’s (2010) idea, it is important to reflect on the whole picture in order to determine how many athletes experience mental health issues *because* of their sport, how many would have suffered from it *anyway* (if they had chosen another profession) and how many are ‘*protected*’ from such issues so long as they stay competing?

## What Does Normal Mean with Non-Normal Populations?

Discriminating between ‘normal’ (healthy) and ‘pathological’ is not always clear when it comes to elite sports (Markser, 2011), perhaps because symptoms encountered by elite athletes might differ from the usual criteria, making mental disorders more difficult to detect (Armstrong et al., 2015; Doherty et al., 2016). Psychiatrists and psychologists are used to diagnosing psychopathological conditions but, to do so, they relate to diagnostic criteria and screening tools originally developed from and for the general population. Without criteria specific to elite performers, nor which consider the sport context in which athletes are progressing, it becomes easy to jump erroneously to the conclusion that a behaviour or an attitude is pathological when the behaviour or attitude in question might be useful and functional in that particular context. Because of an increasing concern regarding mental health problems and their consequences if not appropriately handled, there may be a tendency to “cry wolf” too soon when it comes to elite and, especially, mediatised athletes.

Erroneous diagnoses are often due to a lack of understanding regarding the nuances of an individual’s culture leading to consider as pathological normal variations of his/her behaviour (American Psychiatric Association [APA], 2013). As an example, using anthropometric measures like the Body Mass Index (BMI) or the DSM-V (Diagnostic and Statistical Manual of Mental Disorders, fifth edition) criteria might not be the best indicators when it comes to detect eating-disorder patterns in elites. Athletes are commonly subject to intense physical training, diet restrictions, lower bodyweights, and/or fat percentages than the general population, often independent of any eating disorder or symptoms (Reardon and Factor, 2010; Bär and Markser, 2013). If a lean body is an advantage for performers in many sports (i.e., gymnastics, ski jumping, and long-distance running; Sudi et al., 2004; Thompson and Sherman, 2014), increased body mass is beneficial in others (i.e., open-water long distance swimming, sumo wrestling, linemen in American football; Berglund et al., 2011). So, although weight gain might raise concerns within the general population, adipositas athletica is considered as functional and indispensable in sports where an increased body fat protects, for example, against hypothermia and improves performance (e.g., increased strength, endurance; Berglund et al., 2011).

Of course, because of this focus on weight and body image, eating disorders have been one of the most studied mental health issues in high level performers (Byrne and McLean, 2002; Sudi et al., 2004; Klinkowski et al., 2008; Markser, 2011; Bär and Markser, 2013; Thompson and Sherman, 2014). Yet, discriminating between eating disorders, disordered eating, and context-normal nutrition in such environments remains quite challenging. Eating behaviours might be too quickly seen as symptomatic of an eating disorder instead of as an informed attempt to improve performance (Thompson and Sherman, 2014). Eating disorders or disordered eating are complex and should not be simply viewed as resulting mainly and exclusively from sport participation. Athletes confronted with those kind of issues might have developed them even without performing at a high level or without doing any sport (Thompson and Sherman, 2014). Moreover, in some cases sport participation might actually serve to *prevent* the development of such disorders and become a protective factor by increasing an athlete’s satisfaction with body image (Giel et al., 2016) and self-esteem (Klinkowski et al., 2008). Health professionals must, therefore, be careful. Presence or severity may be misestimated; partly because the sport culture in which elite athletes progress influences the way they live, express and react to mental health issues (Doherty et al., 2016). Alternatively, some athletes might suffer from such disorders, others simply present anorexia-like behaviours or physique while being healthier—psychologically speaking—than people suffering from eating disorders (Klinkowski et al., 2008; Berglund et al., 2011). As a result, both diagnosis and treatment planning become more challenging (Markser, 2011).

In fact, eating disorders are only one of many conditions where normal criteria may be erroneously applied to non-normal people. Differences between elite athletes and the general population can be highlighted by drawing a parallel with medical conditions. A left ventricular hypertrophy is a cardiovascular malformation. Yet, in swimmers, rowers or cyclists, for example, this heart remodelling becomes a functional physiological adaptation due to intense exercise training (Muir et al., 1999; Perseghin et al., 2007). Of course, above a certain threshold, albeit different to the general population warning level, this condition is also considered as pathological for athletes (Muir et al., 1999). This analogy exemplifies a perhaps neglected point, however: namely, that elite athletes are *not* normal so different criteria must be applied. So, while in physiology, a condition called “athlete’s heart” has already been highlighted, we advocate the same distinction regarding “athlete’s psychological state”. Thus, returning to our depression example mentioned earlier, should we be surprised when an individual who has dedicated his/her persona to achievement in a certain field is depressed when he/she fails? Much more relevant is to consider the duration (related to the last game or a chronic state), genesis (from sport, life or both), controllability (clinical or non-clinical, impact on the person), and impact of the condition. Comparison to other highly ego-involved individuals (in music, dance or business, for example) would also seem sensible.

## Functional Versus Dysfunctional Behaviour – it Depends on Context

Expanding this contention, the term “normal” as used with Mr. and Ms. Everybody seems inadequate when talking about elite athletes. Practitioners and, sometimes, researchers have the upsetting habit of seeing everything in black and white (normal *versus* pathological) and often forget that shades of grey exist. Such blurred lines depend on the individual but also on the context. As suggested by Williams (2012), research often neglects the unique characteristics necessary for elite athletes’ lives and fails to understand the decisions they take in a constant effort to stay on top. Behaviours, functional when it comes to elite performers (e.g., weight-restricting behaviours, an apparent lack of work–life balance, excessive training, self-centred ruthlessness), can sometimes be considered as abnormal and look like symptoms associated with mental disorders when compared to the general population (Reardon and Factor, 2010). Difficulty distinguishing between OCD, pre-performance routine and superstitious belief in elite contexts is an example of this thin and blurred line (Swann et al., 2015).

Being an elite athlete “it’s not just a job, it’s a lifestyle, it’s a way of life being an athlete” (Pickering, 2016 – four-time swimming world champion). As such, we suggest replacing the terms “normal” and “pathological” (as used to describe the average population) with the terms “functional” versus “dysfunctional” in high-performance contexts. While they might be seen negatively in everyday life (MacNamara and Collins, 2015), some of those so-called abnormal behaviours and/or attitudes might simply be functional in a high-performance context. A behaviour will, therefore, be seen as functional when beneficial to an athlete’s performance and personal development, and dysfunctional when his/her performance and/or well-being are threatened. Of course, it should also be acknowledged that elite athletes must function as both performer and ‘normal’ individual. As such, the scope of any behaviour, as well as its potential for dysfunctional impact, must be considered.

Reflecting this complexity, caution is required when using this new perspective. *Some* “functional” behaviours can, given the context, become “dysfunctional” (MacNamara and Collins, 2015). Perfectionism and passion, for example, are often considered as positive psychological characteristics in performers but may also adversely affect an athlete’s performance, development and wellbeing when deployed in excess or inappropriately (i.e., over-commitment, obsessive passion; (Hill et al., 2015; MacNamara and Collins, 2015). Just as a lack of certain behaviours might become a problem at some point. Here again, the context in which those behaviours occur needs to be carefully considered. A positive psychological characteristic may become detrimental when extended to the wrong context (e.g., performance environment versus everyday life context). Furthermore, we need to recognise that normative behaviour (what is usually expected in a certain societal group) may encourage actions which are dysfunctional in other contexts. For example, mental toughness in rugby players or aggression in martial artists. Finally, we need to acknowledge that sport expectations may inhibit more general development (e.g., identity foreclosure; Murphy et al., 1996) and perhaps lead to mental health issues in later life.

## Implications for Research and Applied Practice

Our paper highlights several important issues for consideration: Is it appropriate to apply normal population-based diagnostic criteria and screening tools to elite performers? What does normal mean when dealing with a non-normal, non-average population (cf. American Psychiatric Association [APA], 2013)? Is general psychiatric knowledge adequate to effectively diagnose and treat elite athletes (Markser, 2011)? Usual clinical criteria do not apply to non-average people, so clinical screening tools developed for the normal population may not be appropriate for use on elite performers as the performance dimension and its challenges are not considered. Anorexia athletica, adipositas athletica, overtraining syndrome (OTS) and burn-out are some of the few clinical diagnosis that have been created or adapted specifically for athletes, with the intention of taking specific sport-related criteria into account while making a diagnosis (Raedeke and Smith, 2001; Sudi et al., 2004; Berglund et al., 2011). In fact, many conditions and contexts suggest the need for sport-based clinical criteria and screening tools. Meeusen et al. (2006, p. 4) have, for example, suggested that “athletes and the field of sports medicine in general would benefit greatly if a specific, sensitive simple diagnostic test existed for the diagnosis of OTS”. This suggestion is also true for many of the other mental health issues which athletes may encounter, especially when the conditions have a genesis in sport involvement. Burnout has given birth to extensive research in sport (Raedeke and Smith, 2001; Eklund and Defreese, 2015) but, in order to properly investigate this phenomenon, researchers have had to adapt Maslach’s original definition to the sport-specific context: primary symptoms regarding athlete burnout being slightly and subtly different from the ones commonly mentioned in the general population (Raedeke and Smith, 2001). Such diagnostic adaptations further highlight the need to take the sport context and its specificities into consideration while defining the criteria leading to a clinical diagnosis within elite performers.

Reflecting these issues, health professionals must avoid over-diagnosing and falling into the pathologisation trap. In order to make an accurate diagnosis, they must take all the parameters into account (i.e., the symptoms, the context, longevity of the symptoms, their intensity, etc.). Of course, in some cases, athletes’ behaviours or attitudes might be pathological (Thompson and Sherman, 2014) but also the accepted norm in that sport. There is, therefore, an urgent need for qualified and trained practitioners with both clinical *and* sport expertise to deal with the diagnostic and therapeutic challenges relative to mental health in high performance environments.

Furthermore, future research should focus on adapting clinical criteria developed originally from data on the general population to the sport context *and* to specifically develop and validate more sensitive and context-specific screening tools adapted to this unique population (Raedeke and Smith, 2001; Gouttebarge et al., 2015a). Further quality research on mental health in high performance environments will also allow us to proactively support elite and developing athletes on their way to the top, whilst acknowledging and catering for the potentially negative influence of another highly driven but involved individual; namely, the coach. As stated earlier, this reflection is not only valid in sport but also in other performance settings such as music or other performing arts (i.e., dance, drama; Pecen et al., 2016).

## Conclusion

Normal rules do not apply to non-normal people and elite athletes are, by definition, not “normal” in the sense of average. Within this brief review, we have highlighted some issues and challenges resulting from applying general population-based science to non-average people like elite performers. As a consequence, we have tried to move the debate from a normal-versus-pathological point of view to a functional-versus-dysfunctional and person-centred perspective that considers the context in which athletes are progressing.

Of course, if the prevalence of mental health disorders in elite sport should not be maximised, it should not be minimised either. Currently, the prevalence of mental disorders remains unclear and more research is needed in order to evaluate the extent of this issue in elite performers. Both clinical and psycho-behavioural differences between elite athletes and the general community need to be addressed in order to adequately diagnose, treat and prevent the development of mental health issues (Bär and Markser, 2013). This is an important topic but, perhaps, with greater complexity than currently acknowledged. Further research will be needed to respond to the question of our title: “Is elite sport (really) bad for you?”. A causal relationship explaining the occurrence of mental disorders in elite athletes needs to be formally established (Martindale et al., 2014) before to be able to respond to this intricate question.

## Author Contributions

Both authors were involved in the conception and design of the work. FL prepared a draft of the manuscript. DC provided critical revisions to the final submitted version. FL and DC gave approval for it to be published. Finally, all authors agreed to be accountable for all aspects of the work in ensuring that questions related to the accuracy or integrity of any part of the work are appropriately investigated and resolved.

## Conflict of Interest Statement

The authors declare that the research was conducted in the absence of any commercial or financial relationships that could be construed as a potential conflict of interest.
